# Public Aspects of the Prevention of Consumption

**Published:** 1906-12-08

**Authors:** 


### PUBLIC ASPECTS OF THE PREVENTION OF CONSUMPTION.

IN an excellent address on the public aspects of the prevention of consumption Dr. Philip of Edinburgh declares as the text for his thesis that "If communities, as communities, are to benefit practically by the discovery of the tubercle bacillus to the extent which the discovery warrants, there is clamant need for a vastly wider organisation and co-ordination of measures than has yet been realised." He then proceeds to discuss what these measures should be, and gives a full description of the way in which, largely due to his own personal influence and work, they have been actually carried out in the city of Edinburgh.

The first measure which he regards as an essential preliminary, to any adequate attempt on the part of public bodies to cope with the disease, is compulsory notification. As he argues, all the difficulties which have from time to time been suggested in relation to the practical carrying out of notification, were similarly advanced in connection with the proposed notification of other infectious diseases, and, indeed, in relation to other State and municipal procedures, but these difficulties rapidly melt away in practice. Compulsory notification has been successfully carried out in Norway and the United States. The Local Government Board of Scotland has recently expressed itself in favour of a system of notification, and points out that pulmonary phthisis is an infectious disease within the meaning of the Public Health Act. No further legislative measures are therefore necessary for the introduction of compulsory notification of this disease. Some form of notification has already been established in Manchester, Leeds, Bradford, Liverpool, and Brighton, while Sheffield instituted compulsory notification two years ago. By such means definite knowledge is obtained of the extent of the disease in the community, and its foci of infection, and the question then arises as to suitable measures for the treatment and disposal of the varying elements of these foci of infection.

For this purpose Dr. Philip conceived the idea of a tuberculosis dispensary, and as far back as 1887 founded the Victoria Dispensary for Consumption in Edinburgh. The objects of this institution are briefly enumerated as follows:—

1. The reception and examination of patients at the dispensary, where a detailed record of each case is kept.

2. The bacteriological examination of expectoration and other discharges.

3. The instruction of patients how to treat themselves and how to prevent infection to others.

4. The dispensing of medicines, sputum bottles, disinfectants, and even foodstuffs in suitable cases.

5. The visitation of patients at their own homes by (1) a qualified medical man, and (2) a specially trained nurse for the double purpose of treatment and of investigation into the conditions of the dwelling.

6. The selection of suitable cases for the hospital, sanatorium or incurable home, and the supervision of patients after discharge from the hospital.

7. The guidance generally of tuberculous patients and their friends to answer inquiries from all interested persons on every question concerning tuberculosis.

From this outlined programme it will be seen that the dispensary forms a centre of active campaigning against the inroads of the disease in the community, and affords also a valuable means of accumulating a mass of statistics and information regarding the incidence of the disease in the district. In connection with such a dispensary Dr. Philip would have:

1. A hospital for advanced or dying patients.

2. A sanatorium for selected persons with a view to cure.

3. Tuberculosis colonies to which could be drafted patients in whom the disease has been more or less arrested, but for whom a return to ordinary occupations would probably lead to relapse.

After much pleading, Dr. Philip has succeeded in securing arrangements for the reception of 50 cases of advanced consumption at the New City Hospital, Colinton Mains, and there is also the Royal Victoria Hospital for Consumption as a sanatorium for curable cases. With the same persistence and enthusiasm which he has hitherto shown, he will probably succeed in completing his scheme by the institution of these proposed tuberculosis colonies. Dr. Philip is satisfied that such colonies could be made self-supporting. The residents on the colony would be recruited by selection from patients whose disease had been successfully treated at the sanatorium. The workers would remain at the colony under medical surveillance, and the character and amount of their work would be suitably gauged and directed. The produce of the colony resulting from the patients' labours would form an important supply for the sanatorium.

Dr. Philip is very emphatic in maintaining that sanatoriums should be founded in immediate relationship to the large centres. He points out that it is a prevalent but erroneous belief on the part of the public that a cure can only be effected under conditions which their ordinary residence and station in life will not permit them to enjoy.

The essential feature of this scheme is that each institution shall form a part of one complete organisation, of which the component parts shall be intimately connected one with each other, the dispensary forming the central pivot of the whole.

